# Data Analytics to Support Policy Making for Noncommunicable Diseases: Scoping Review

**DOI:** 10.2196/59906

**Published:** 2024-10-25

**Authors:** Giorgos Dritsakis, Ioannis Gallos, Maria-Elisavet Psomiadi, Angelos Amditis, Dimitra Dionysiou

**Affiliations:** 1 Institute of Communication and Computer Systems National Technical University of Athens Athens Greece; 2 Directorate of Operational Preparedness for Public Health Emergencies Greek Ministry of Health Athens Greece

**Keywords:** policy making, public health, noncommunicable diseases, data analytics, digital tools, descriptive, predictive, decision support, implementation

## Abstract

**Background:**

There is an emerging need for evidence-based approaches harnessing large amounts of health care data and novel technologies (such as artificial intelligence) to optimize public health policy making.

**Objective:**

The aim of this review was to explore the data analytics tools designed specifically for policy making in noncommunicable diseases (NCDs) and their implementation.

**Methods:**

A scoping review was conducted after searching the PubMed and IEEE databases for articles published in the last 10 years.

**Results:**

Nine articles that presented 7 data analytics tools designed to inform policy making for NCDs were reviewed. The tools incorporated descriptive and predictive analytics. Some tools were designed to include recommendations for decision support, but no pilot studies applying prescriptive analytics have been published. The tools were piloted with various conditions, with cancer being the least studied condition. Implementation of the tools included use cases, pilots, or evaluation workshops that involved policy makers. However, our findings demonstrate very limited real-world use of analytics by policy makers, which is in line with previous studies.

**Conclusions:**

Despite the availability of tools designed for different purposes and conditions, data analytics is not widely used to support policy making for NCDs. However, the review demonstrates the value and potential use of data analytics to support policy making. Based on the findings, we make suggestions for researchers developing digital tools to support public health policy making. The findings will also serve as input for the European Union–funded research project ONCODIR developing a policy analytics dashboard for the prevention of colorectal cancer as part of an integrated platform.

## Introduction

### Noncommunicable Diseases as a Public Health Challenge

Noncommunicable diseases (NCDs), such as cardiovascular or chronic respiratory diseases, cancer, and diabetes, account for 74% of all deaths globally [1]. In the European Union (EU), NCDs are responsible for almost 80% of the disease burden and most premature deaths [2]. They affect quality of life and life expectancy, create numerous challenges for patients and their families, and have a large financial impact, costing EU economies more than 100 billion US$ annually. As many NCDs are age-related, their burdens are increasing partly due to the prolonged lifespan of the population [3]. NCDs are strongly associated with a number of preventable factors, such as smoking, physical inactivity, harmful alcohol use, and unhealthy diet, and environmental factors, such as air, water, soil pollution, and chemical exposure. Interventions for controlling such risk factors and promoting health and well-being have the potential to reduce the prevalence of NCDs by as much as 70% [4]. To this end, the European Commission has launched an initiative to support effective policies and actions to reduce the burden of major NCDs and improve citizens’ health and well-being [2].

The successful management of NCDs requires the integration of the best available scientific evidence into decision-making [5]. Effective use and reporting of data can guide the process and empower policy makers to better understand and act [6]. On the other hand, research findings that directly apply to the policy of interest, when available, are often inconsistent, out of date, or of poor quality. As a result, policy making is traditionally based on social context, political agendas, expert opinion, or the media, all of which are usually biased [7]. Currently, this traditional policy-making approach, which abides more to the notion of clinical health services delivery, is simultaneously challenged from various aspects as it appears unable to meet the novel needs of decision makers [8]. From population surveillance data and indicators to big data and time-tagged trends, policy makers’ informed decisions nowadays predispose the effective deployment of technological advancements regardless of whether they concern NCD screening and treatment or managing emerging crises. The recent COVID-19 pandemic signaled the transition into the digital health era, where a newfound model of supporting policy makers’ decision processes is needed [9].

### Harnessing Digital Technologies for Public Health Policy Making

In the last 2 decades, the growth of health care data in quantity and complexity and the rapid advances in the fields of big data analytics (BDA) and artificial intelligence (AI) present an opportunity to transform conventional policy making into a data-driven process, utilizing various health-related data sources such as electronic health records (EHRs), public health databases, and social networking platforms [10-12]. The integration of routinely collected real-world data and use of advanced analytical techniques for improved decision-making is also referred to as “precision public health” as opposed to traditional public health [13].

Reliable data, combined with data analytics, play key roles at various stages of the public health policy-making process by aiding in understanding, priority setting, resource allocation optimization, identification of the optimal intervention, implementation, and evaluation [14]. This capability allows policy makers to base their decisions on empirical evidence by identifying patterns, trends, and correlations that might not be evident otherwise. Such informed decision-making is vital in areas like health care and can lead to more effective outcomes [10]. Specifically, data analytics may improve the delivery of public services, enabling governments to anticipate public health crises, optimize public health initiatives, increase adherence from the public, and enhance the overall responsiveness of services [15]. This iterative process of policy evaluation and adjustment, supported by real-time data monitoring, ensures that strategies are continuously refined to achieve intended goals. Analytics can identify high-impact areas for efficient resource allocation, such as targeting aggressive policies where they are most needed (eg, the outbreak of a pandemic).

Data analytics approaches are commonly categorized into 3 broad types: descriptive, predictive, and prescriptive analytics. The 3 types answer different questions but use similar methodologies that can be applied individually or in combination, depending on the health policy objectives, data availability, and decision-making context. Descriptive analytics summarize past and present trends and patterns in the data to answer the question “What happened?” Predictive analytics use primarily historical data to create models that answer the question “What will happen?” Prescriptive analytics employ data-driven models and optimization algorithms to recommend the most effective actions, interventions, or allocation strategies trying to answer the question “What should I do?”

The use of data analytics in the context of policy making has also been referred to as “policy analytics” by some authors [16]. It has been suggested that policy analytics include data-driven tools that respond to a policy need and use a transparent development process [17]. Examples of policy analytics techniques are statistics, simulations, data mining, machine learning (ML), social networks, and geographic info [18].

### Rationale and Aims

Currently, there is no comprehensive overview of the use of data analytics tools to support policy making for NCDs. Such an overview could guide stakeholders and organizations involved in policy making, engineers and companies managing such tools, data analysts, and researchers, and could highlight gaps and unmet needs in the area. The present review has been performed in the context of the Horizon Europe project ONCODIR [19]. The project seeks to identify risk factors associated with colorectal cancer (CRC) and will integrate multidisciplinary research methods and technologies to deliver evidence-based and personalized recommendations on CRC. The findings of this review will serve as an evidence base for the development of the policy analytics component of ONCODIR’s integrated platform.

The primary aim of this scoping review is to investigate the landscape of data analytics tools and platforms designed to support evidence-based policy making for NCDs. The secondary aim is to explore the adoption of these tools by policy makers and the factors affecting this.

## Methods

### Design

Α scoping review was selected given the aim of this study. It has been suggested that scoping reviews are more suitable than traditional systematic reviews and meta-analyses when focusing on rapidly evolving topics, as they allow more timely synthesis of evidence. The inclusive and broad nature of scoping reviews also makes them better at looking into the range of evidence on a particular topic [20]. We conducted our review according to the Joanna Briggs Institute guidelines to ensure quality and reliability [21]. We followed the PRISMA-ScR (Preferred Reporting Items for Systematic Reviews and Meta-Analyses Extension for Scoping Reviews) guidelines (Multimedia Appendix 1) and developed a search strategy based on the Population, Concept, and Context framework as follows [22]:

- Population: humans with NCDs
- Concept: digital data analytics tools
- Context: public health policy making

Based on this framework, in December 2023, we searched the PubMed database for publications written in English from 2013 to date. The search was repeated in July 2024. We searched for articles published in the last decade to explore the latest advances in the field. We searched for original research and review articles published in peer-reviewed journals and no other sources to ensure the highest quality of the studies.

A query was first developed in PubMed and was then adapted for IEEE Xplore: ((“health”[Title/Abstract] OR “healthcare”[Title/Abstract] OR “clinical”[Title/Abstract] OR “medical”[Title/Abstract]) AND (“policy”[Title/Abstract] OR “policies”[Title/Abstract] OR “policymaking”[Title/Abstract] OR “policy-making”[Title/Abstract] OR “policy making”[Title/Abstract] OR “decision making”[Title/Abstract] OR “decision-making”[Title/Abstract] OR “decision maker”[Title/Abstract]) AND (“analytics”[Title/Abstract] OR “analytic”[Title/Abstract]) AND (“data”[Title/Abstract] OR “evidence”[Title/Abstract] OR “evidence-based”[Title/Abstract])).

Given that the concept of policy analytics and the associated terminology are not yet well defined, we chose broad terms in the first place to widen the results and assessed eligibility later case by case. For instance, we did not use a term for NCD as it appeared that usually the names of the individual conditions are used. Moreover, we preferred “health” as “public health” is not consistently used in the context of policy making for NCDs.

### Study Selection

Title and abstract screening and full-text review were performed according to predefined eligibility criteria (Textbox 1). A snowballing approach was also used to identify any additional articles from the reference lists of the screened articles. We included studies describing data analytics tools or their use in detail. These had to be concrete tools or applications designed specifically with the aim to support policy makers as opposed to models, algorithms, or other theoretical frameworks, such as decision-analytic models or cost-effectiveness analyses. Analytics tools had to be designed for or applied to a specific NCD as these have been defined according to the World Health Organization (WHO) [23]. We excluded analytics not performed on specific conditions or not applied to health data (eg, health care management). We also excluded studies on views or perceptions about analytics.

From all included articles, we extracted data related to the disease studied, purpose of the study and tool, analytics used, implementation, and link with policy making. We performed a narrative, descriptive data synthesis of the techniques used and their implementation.

**Eligibility criteria for article selection.**

**Inclusion criteria**

- Original research or review.
- Study on humans with noncommunicable diseases (NCDs).
- The study includes a concrete digital data analytics tool that can be used by a policy maker.
- The study includes a tool that is designed to support health policy making.
- The study includes analytics performed on health-related data.

**Exclusion criteria**

- Commentaries, viewpoints, protocols, perspectives, or other study types.
- Study on infectious diseases or no disease (eg, health management).
- The study describes theoretical models, frameworks, or statistical algorithms.
- The study includes tools for clinical decision support, patient benefit, or other purposes.
- The study includes analytics performed on the literature.

## Results

### Study Characteristics

**Figure 1:**
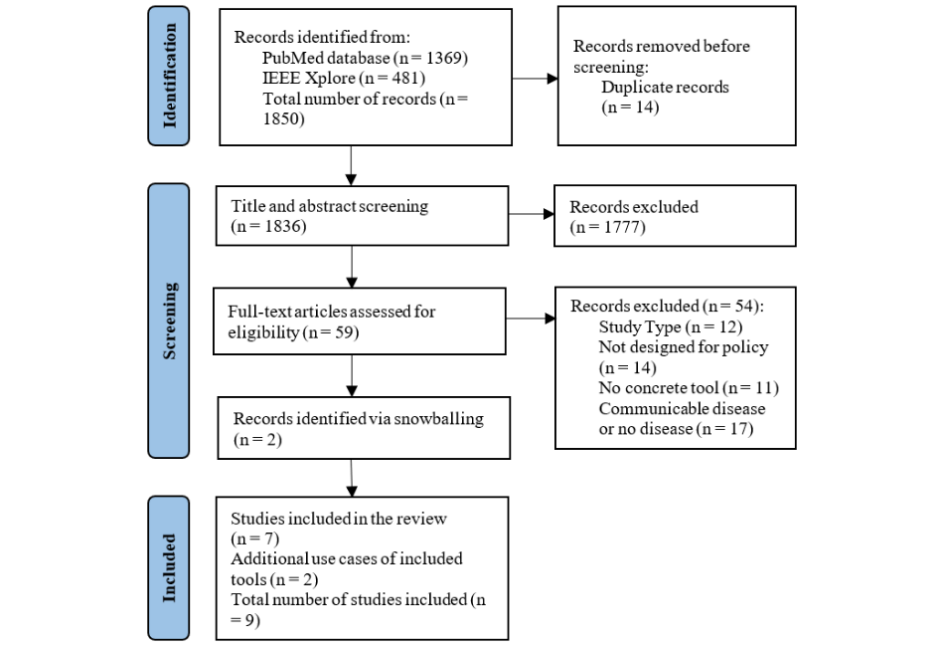
PRISMA (Preferred Reporting Items for Systematic Reviews and Meta-Analyses Extension for Scoping Reviews) flow diagram of the stages of the scoping review.

The search yielded 1850 articles from the PubMed and IEEE Xplore databases (Figure 1). After article duplicates were removed, 1836 remained for title and abstract screening. A total of 59 articles were identified for full-text review. Five studies met our eligibility criteria and were included in the review. Two more studies were identified via snowballing of the reference lists of screened articles. Furthermore, 2 articles were added that included additional applications of the identified tools, as one of the aims of the review was to explore implementation and uptake. In total, 9 articles were included in this review. Articles were published between 2017 and 2022. The articles described 7 data analytics tools or integrated platforms including a data analytics component. The studies described the tools in detail and included use case scenarios, pilot studies, workshops, and examples of implementation that illustrate how analytics can be used to support policy making for NCDs. Four studies were conducted in the United States, 1 in Australia, and 1 in Canada. Three studies were conducted in various countries of Europe (ie, Greece, Germany, Slovenia, Spain, United Kingdom, Sweden, Finland, Northern Ireland, Republic of Ireland, and Denmark).

### Tools Overview

The 7 tools reviewed are summarized in Table 1. Three integrated platforms (EVOTION, MIDAS, and CrowdHEALTH) were designed to support public health policy decisions for a range of conditions and include a data analytics component supporting both descriptive and predictive analytics [24-28]. Users can create policy models, define the way in which data should be analyzed in order to produce the evidence useful for public health policy making, and obtain analytical results of how this evidence may support or contradict various policy actions. The other 4 tools (PoPHQ, PoPHR, Social InfoButtons, and RiskScape) were designed for disease monitoring through the integration, descriptive analysis, and visualization of population health data from various sources [29-32].

Two of the 7 tools were designed to address specific NCDs. EVOTION was specifically designed for hearing loss and PoPHQ was designed for obesity prevention. The other platforms were applicable to and piloted with a wide range of conditions, including obesity in adults and children, cardiovascular conditions, chronic kidney disease, chronic respiratory conditions, cancer, mental health, asthma, and diabetes. Three platforms were piloted in obesity, physical activity, and nutrition. Two platforms were piloted with each of the following conditions: respiratory, cardiovascular, and diabetes. CrowdHEALTH was piloted with cancer [25]. Six of the 7 tools were designed for or used with NCDs only, while PoPHR was applicable to infectious diseases as well [30].

**Table 1:** Overview of the tools included in this review.

Tool	Type	Analytics supported	Implementation reported	Study
EVOTION	Public health policy decision support	Descriptive and predictive	Analytics applied, use case example, and workflow	Prasinos et al [27] and Saunders et al [28]
CrowdHEALTH	Public health policy decision support	Descriptive and predictive	Use case example, workflow, and use cases including health care stakeholders	Moutselos et al [26] and Mavrogiorgou et al [25]
MIDAS	Public health policy decision support	Descriptive and predictive	Pilots with policy makers	Shi et al [24]
PoPHQ	Health informatics tool	Descriptive	Design workshops where stakeholders identified user stories and use cases	Canfell et al [29]
RiskScape	Public health surveillance	Descriptive	Real-life implementation	Canfell et al [29] and Cocoros et al [32]
Social InfoButtons	Public health analytics	Descriptive	Use case scenarios	Ji et al [31]
PoPHR	Visualization of population health data	Descriptive	Description and functionalities with examples, and initial implementation ongoing	Shaban-Nejad et al [30]

### Study Settings

Details of the use cases are provided in Table 2. The studies presented the 7 tools using different settings, including descriptions of the functionalities with examples, use case scenarios where authors presented a workflow, pilot studies with actual data from various sources, applications of analytics on data collected using the tool, design workshops with stakeholders, and real-life implementation.

CrowdHEALTH was piloted in 5 different countries and 6 use case scenarios with stakeholders from various health care organizations [25]. Relevant data from patients and healthy adults and children were used to address policy needs on various conditions. For example, the Slovenian pilot used data on physical activity to analyze the physical fitness and weight status of children, assess its development over time, and predict future levels. This provided a basis for the implementation of policies that link school and health data for early intervention monitoring and evaluation. Interestingly, this use case was based on a real-life policy described in the Slovenian National Program on Nutrition and Physical Activity for Health 2015-2025 [26]. MIDAS was piloted in 4 countries using social media, MEDLINE analytics, and news media data on a range of conditions [24]. Ji et al [31] used data on treatments and symptoms posted by patients on social media to evaluate the effectiveness of the Social InfoButtons platform. PoPHR was piloted with a randomly sampled, open cohort of 25% of the Montreal population in Canada [30]. PoPHQ is currently in the mock-up phase but has been designed to integrate anonymized electronic medical record data from Queensland, with a final total sample size of approximately 5 million [29]. No pilot data were reported for Risk Scape, but the Massachusetts Department of Public Health (MDPH) is currently using the platform to monitor conditions of interest using EHR data that are updated monthly from clinical practice groups that cover approximately 20% of the state population [32]. Prasinos et al [27] described the workflow of policy creation, selection, and execution of analytics using the EVOTION platform. Saunders et al [28] reported 3 applications of BDA techniques using a dataset synthesized from the EVOTION data repository.

**Table 2:** Details of the analytics included in this review.

Study and country			Noncommunicable disease		Analytics		Purpose		Data
**Prasinos et al [27]**									
	Greece, United Kingdom, and Denmark	Hearing loss (HL)		Basic statistics Linear regression Principal component analysis Inferential statistics		Investigate the impact of hearing interventions on quality of life (QOL)		Synthetically generated dataset from the EVOTION data repository	
**Saunders et al [28]**									
	Greece, United Kingdom, and Denmark	HL		Predictive modeling Regression analysis Generalized linear mixed model Correlations		Estimate the risk of noise-induced HL Predict hearing aid (HA) usage from changes to the sound environment Examine the association between physical activity and HA usage		Synthetically generated dataset from the EVOTION data repository	
**Moutselos et al [26]**									
	Slovenia	Obesity		Forecasting Simulation Causal analysis		Effectiveness of various interventions for obesity prevention Early detection of children with increased risk linked to poor physical fitness		Large-scale data from the national surveillance system on the physical and motor development of children	
**Mavrogiorgou et al [25]**									
	Spain	Obesity in adults		Clinical pathway mining Risk stratification		Identification of overweight patients		Demographic information, hospitalization, emergency and hospital episodes, and morbidity	
	Sweden	Cardiovascular and chronic kidney disease		Clinical pathway mining Risk stratification Causal analysis		Monitor patients		Demographic, drug usage, and practitioners’ consultation data	
	Slovenia	Fitness and obesity in childhood		Clinical pathway mining Risk stratification Causal analysis		Analyze physical fitness and weight status Predict future levels of fitness and somatic development		Physical activity, sedentariness, sleep, heart rate, socioeconomic status, and parental physical activity	
	Greece	Chronic respiratory conditions		Risk stratification		Monitor disease progression and health care expenditure for improved chronic disease management of patients		Biosignals from pulse oximeters, blood pressure meters, glucometers, spirometers, weighing scales, and activity trackers	
	Germany	Nutrition and physical activities		Clustering analysis Correlation analysis		Understand the influence of nutritional habits and physical activity on overall health and QOL		Physical and activity data provided by activity trackers	
	Greece	Cancer care		Causal analysis		Evaluate the impact of online coaching and medical education		Diagnosis, treatment, comorbidities, health behaviors, and side effects	
**Shi et al [24]**									
	Spain	Obesity		Random forests/least absolute shrinkage and selection operator		Identify the risk factors of childhood obesity		Controlled and open data including social media analysis, MEDLINE analytics, and news media analysis	
	Finland	Mental health		Lexis diagram analysis Descriptive analysis		Aggregate, summarize, and visualize risk factors Evaluate health, social, and educational status		Controlled and open data including social media analysis, MEDLINE analytics, and news media analysis	
	Northern Ireland	Social care for children		Markov chain Long short-term memory (LSTM) network		Track patterns of behavior over time Estimate the probability of transition between different types of care Improve the protection of children		Controlled and open data including social media analysis, MEDLINE analytics, and news media analysis	
	Republic of Ireland	Diabetes		Autoregressive integrated moving average (ARIMA) model		Forecast the consumption of diabetic drugs		Controlled and open data including social media analysis, MEDLINE analytics, and news media analysis	
**Canfell et al [29]**									
	Australia	Obesity		Comparison across age groups Counts by facility Counts and percentages Stratification by suburb and facility		Target interventions across the life course Direct resources Justify the problem Compare obesity across regions		“Mock-up” without patient data^a^	
**Canfell et al [29] and Cocoros et al [32]**									
	United States	Diabetes, hypertension, asthma, and obesity		Heat maps by zip code Stratification by demographics and comorbidities Time series analyses with trend statistics Data aggregation and visualization		Review, analyze, map, and trend aggregate data Prevalence of selected conditions Identify health disparities		Electronic health records (EHRs) from 3 clinical practice groups that cover approximately 20% of the state population (>1.2 million)	
**Ji et al [31]**									
	United States	Posttraumatic stress disorder and asthma		Statistical Geospatial Temporal Topic investigation Association discovery Recommendation discovery Visualization		Compute statistical aggregates Explore data according to a geographic feature Analyze trends over time Explore correlations between treatments, side effects, symptoms, and conditions Discover treatment recommendations Integrate openly available health data		Openly available health data sources including SMN, Twitter, MedHelp, WebMD, CDC, and PubMed	
**Shaban-Nejad et al [30]**									
	Canada	Chronic diseases (eg, diabetes, hypertension, coronary heart disease, and stroke)		Visualization Stratification Filtering Statistical algorithms to detect changes in an indicator over time and space		Explore and visualize available indicators Create coherent portraits of population health and health system performance Evaluate the effects of public health interventions		No actual data used in this study; initial implementation ongoing^b^	

^a^Final total sample size is estimated to be approximately 5 million (anonymized electronic health record data from Queensland).

^b^Initial implementation with a randomly sampled, open cohort of 25% of the population of the Census Metropolitan Area of Montreal, Quebec; in the process of implementation in the entire population of the province of Quebec.

### Data Analytics Applied

Table 2 presents the data analytics applied in detail, including the setting, purpose, data used, NCD studied, and country where the study took place. The choice of analytics is strongly linked to the policy need being addressed and the nature of the tool. Α wide range of analytical techniques were used, which can be summarized as follows.

#### Descriptive Analytics

All tools employed descriptive analytics. The first level was data ingestion, integration, cleaning, and preprocessing, such as removal of duplicates and errors, imputation of missing data, handling of outliers, and standardization of data formats [24,25,30,31]. The next step was data exploration using basic descriptive statistics and inferential statistics, such as the identification of risk factors for a specific condition. Static or interactive visualizations were used, including scatter plots, heat maps, bar or box plots, and pie charts. Three of the tools employed more specialized techniques, including geospatial analytics or mapping (ie, exploration of data in a geographical area), temporal analytics (ie, tracking trends over time), and comparative analytics, which identifies differences between groups of measurements, such as disease prevalence across different age groups [29,30,32]. Other types of analyses used were clustering analysis (ie, grouping of objects based on measures of similarity) and correlation analysis to identify the strength of the linear association between variables. All tools included a visualization dashboard or user interface, although there were differences in the type or level of user interaction.

#### Predictive Analytics

Three out of the 7 tools employed predictive analytics. Methods included regression analysis and statistical modeling. Contemporary frameworks were also used for prediction and forecasting, including ML, deep learning, and simulation modeling [24,28]. Other types of analyses that were used for predictive purposes included risk stratification analysis; causal analysis, which models the behavior of the target variable of interest; and clinical pathway analysis, which models the process followed during treatment of a patient with respect to a particular condition [25,26].

#### Prescriptive Analytics

No study implemented a concrete prescriptive methodology. Three out of the 7 tools were decision support systems designed to make policy recommendations. The authors referred to the prescriptive capabilities of the tools and demonstrated the policy creation process [24,27,29]. However, none of them presented a related use case actually applying this type of analytics.

### Tool Implementation

Out of the 7 platforms reviewed, 1 has been fully implemented in real life. RiskScape is used by MDPH to monitor conditions of interest using EHR data updated monthly from 3 clinical practice groups that cover approximately 20% of the state population [32]. It has a key role in demonstrating the need and burden for MDPH’s applications for funding through the identification of inequitably burdened populations. The authors suggest that the platform unloads analytical burden from health departments, centralizes information in an efficient electronic environment, and offers clinical practices a holistic understanding of disease patterns and management practices. RiskScape is an open-source software and is publicly available [33]. Shaban-Nejad et al [30] reported that PoPHR had been initially implemented and was in the process of being fully implemented in Montreal, Quebec. According to the authors, the platform can be used by policy makers to improve decisions related to the planning, implementation, and evaluation of population health and health system interventions.

For the remaining 5 tools, the reviewed studies included analytics examples, use cases, or pilots. MIDAS was evaluated by policy makers in the pilot studies and successfully achieved all key progress indicators [24]. The platform received positive feedback on its capacity to integrate and analyze data. The pilots demonstrated how custom-tailored analytics produced knowledge and results that are actionable by public health policy makers and gave them insights for possible future interventions. Based on these results, the authors concluded that MIDAS is transferable, sustainable, and scalable across policies, data, and regions. Mavrogiorgou et al [25] reported how each use case of the CrowdHEALTH platform provided insights that can be used in policy making. Stakeholders from various sectors attended a workshop and provided feedback on the purpose, interface, and overall design of PoPHQ [29]. They identified various uses of the platform to create stories for 4 different end users: public health practitioners, systems planners, researchers, and generic users. PoPHQ is planned to be implemented in Queensland with a population of 5 million [29]. Saunders et al [28] reported that a policy maker could use EVOTION analytics as evidence to expand guidelines aimed at preventing noise-induced hearing loss or simulate hearing aid uptake and usage if urban planning organizations were to project an increase in everyday acoustic noise due to changed requirements for official noise prevention initiatives. Finally, Social InfoButtons can be used by governments for disease surveillance [31].

## Discussion

### Data Analytics for Public Health Policy Making

This scoping review was conducted to explore the data analytics tools designed for public health policy making for NCDs and their implementation. The review was motivated by the emerging need for approaches harnessing BDA, AI, and other novel technologies to improve public health policy making. It was also motivated by the EU-funded research project ONCODIR developing a policy analytics dashboard for the prevention of CRC as part of an integrated platform.

We presented 2 different types of tools enabling data analytics for policy making for NCDs: (1) tools designed for public health monitoring and surveillance that aggregate openly available data or data from electronic medical records and have mainly descriptive analytics and visualization functionalities and (2) integrated platforms designed for policy decision support with both descriptive and predictive analytics functionalities. Previously, Canfell et al [13] reviewed the use of real-world data for precision public health in NCDs and identified surveillance platforms integrating descriptive, comparative, and geospatial analytics. Our review, with its different scope, extends these findings and further demonstrates that predictive analytics can be used for the management of NCDs to inform policy decisions. A variety of ML techniques were used in the studies included in this review for forecasting. Moreover, classic statistical methods, such as logistic regression analysis, were adopted. ML techniques are increasingly used with large population health datasets to improve public health surveillance, disease prediction, and delivery of interventions [34]. On the other hand, prescriptive analytics provide actionable recommendations to policy makers and can have a key role in precision public health. It must be noted here that even though 3 of the platforms included in this review were designed as policy decision support tools, no prescriptive analytics were actually applied in the pilot studies or use cases that generated policy recommendations. Instead, predictive models, risk estimation, and forecasting provided insights that aimed to support policy makers in making decisions. We could claim that the 3 tools reviewed, which employ predictive analytics, are more advanced toward supporting policy decisions compared to tools with descriptive analytics capabilities only. However, the actual usefulness of a tool for policy makers depends on the specific policy need and scenario.

### NCDs Studied

Among the 4 major types of NCDs, cancer appears to be the one that is least studied in the context of data analytics for policy making as only the CrowdHEALTH platform was piloted with cancer. CrowdHEALTH used data from a web platform related to patients’ diagnosis, treatment, comorbidities, health behaviors, and side effects to assess the impact of online coaching and medical education and predict future behaviors of cancer patients [25]. The type of cancer supported by the platform was not specified. According to the authors, given the absence of specific policies for the provision of medical information and online coaching and the increased patient support needs, such an approach may be useful for the improvement of resource allocation in the health care system among others. Other studies have explored protocols for mapping breast cancer registry data [35], the use of modeling to optimize cancer screening and predict catchment areas, and the use of AI for risk stratification of cancer patients [36-38]. However, none of these studies included tools designed to be used by policy makers. To the best of our knowledge, no policy-making platform with an analytics component has been designed for or used with cancer.

To address this gap, the EU-funded ONCODIR research project aims to develop an intelligent policy analytics dashboard as part of an integrated platform to support the primary prevention of CRC. The dashboard will incorporate retrospective data on CRC incidence, risk factors, and other relevant data as well as prospective data from a mobile app, and will enable descriptive and predictive analytics to provide insights to inform CRC prevention policies.

### Use of Tools by Policy Makers

Our findings show very limited real-world use of analytics by policy makers. This is in line with previous studies showing limited implementation of digital tools for NCDs [13]. Only RiskScape is fully implemented and is also publicly available to use. Shaban-Nejad et al [30] reported that PoPHR had been initially implemented and was in the process of being fully implemented in Montreal, Quebec, but no more reports have been published since then [30]. In a subsequent study from 2020 not included in this review, it was reported that PoPHR was in the process of being deployed in Quebec for routine use by public health professionals [39]. In the same study, the authors reported their plans to extend the use of PoPHR to recommend interventions that are likely to be the most effective.

For most of the other tools’ use cases, pilots or evaluation workshops were reported that involved policy makers. For instance, Saunders et al [28] reported some applications of analytics using the EVOTION platform with implications for policy makers [28]. In another study not included in this review, Dritsakis et al [40] reported a series of workshops where EVOTION was demonstrated to stakeholders in 4 countries and evaluated using a Strengths, Weaknesses, Opportunities, and Threats methodology [40]. The study highlighted the potential of the tool together with obstacles and risks that need to be addressed, such as the complicated mechanism of data collection and analysis and the lack of major analytic capabilities required for public health policy decision-making (eg, economic evaluation).

Overall, the 7 tools included in this review have been mostly designed as research prototypes in academic settings. Policy makers were involved in the development or use in some way, and all studies highlight the potential use of analytics to support policy making. However, there is very limited uptake or plans for use by policy makers reported in the reviewed studies. This has implications for the usefulness of the tools as most of them have not been tested in real-life settings. RiskScape (fully implemented) and PoPHR (initially implemented) are currently the most mature data analytics tools that can aid policy making according to the findings of this review. The fact that most tools were developed in the last 4 years could explain the poor uptake to some extent as the tools may not be implemented yet or the implementation may not have been published. Finally, it must be noted that besides RiskScape, it is unclear if the rest of the platforms are accessible and available for use.

### Adoption of Digital Health Technologies

Many studies have explored factors influencing the adoption of digital health technologies by policy makers. Innovative solutions are incorporated into health policy functions at a slower pace than health transformation into a digital asset [41]. Lack of advanced infrastructure, low interoperability levels among critical actors, and bureaucracy pose barriers to the acceptance of new technologies that are frequently exacerbated by safety concerns. Other challenges are organizational fragmentation creating siloed data systems, difficulty in data sharing due to privacy and security issues, and concerns around data quality [42]. Moreover, systemic and organizational issues exist owing to the core characteristics of public health authorities as administrative bodies that lack regulatory frameworks and a data governance culture as a whole [43].

A very important obstacle from the perspective of end users is often the poor IT literacy and lack of digital skills by policy makers and public health professionals [44]. Another aspect is whether these tools actually meet the needs of end users. For example, reviews on the use of visual analytics in mental health care planning have shown that despite the availability of advanced visualization tools, such as geographical maps, the majority of experts use simple, familiar, and readily available visualizations, and a very small percentage of digital tools are actually used for policy and planning [45,46]. Despite a clear need to extract information from highly complex data, such barriers and concerns hinder the use of analytics as part of decision-making and lead policy makers to use approaches that are most familiar to them and widely understood, and keep relying on expert opinion and intuition.

Poor uptake of evidence-based tools is also related to challenges inherent in the policy-making process. Moving evidence into practice will always require political engagement and therefore will be influenced by political agendas [28]. Moreover, there will always be urgent problems and limited funds that will require policy makers to use economics, statistics, and scientific skills to rapidly interpret evidence and provide solutions as the recent COVID-19 pandemic showed. The successful use of data-driven tools for responsive and accurate public health decisions for NCDs requires the optimization and reorganization of the public health sector and workflows [13]. Some priorities that have been reported include investment in modern data management infrastructure, development of strategic partnerships, need for AI transparency and reproducibility, and explicit consideration of equity and bias [47]. To effectively use new technologies and the large amount of health care data that are now becoming available to guide policy decisions, it is necessary to overcome the computational, algorithmic, and technological obstacles of an extremely heterogeneous data landscape, as well as a variety of legal, normative, governance, and policy limitations. Moreover, the transition into the digital health era requires a digital health background and key IT skills to ensure that policy makers have the capacity to make the most out of digital health innovations. A paradigm shift is required for policy makers at local, regional, and national levels to overcome cultural barriers that influence their acceptance and implementation of novel technologies [48].

### Recommendations

Moving forward, we recommend that researchers developing advanced data analytics tools for policy making should engage with end users throughout the process, respond to their needs, and present results in a way that is easy to interpret. They should specify the type and purpose of analytics, how policy makers can use it, and what they can achieve with it. They should also secure the required resources to make the tool available and sustainable after it has been launched. Finally, developers must promote the implementation of digital health tools for decision-making as this could positively influence the IT literacy of policy makers. On the other hand, public health professionals should be encouraged by the potential of data analytics tools to inform evidence-based decisions and empower their digital health skills to be able to use the developed IT tools. Overall acceptance of novel technologies in domains and processes where current uptake is limited will facilitate the required paradigm shift.

To unlock the full potential of data analytics, authorities need to prioritize robust data infrastructure, interoperability, data integration, and the initiation of high-quality nationwide data registries (eg, cancer registries) to monitor public health. Training programs for data governance capacity building, hiring of specialists to key positions, and international collaborative efforts on major public health threats could help transition to more informed and responsive public health policy making. Finally, a strong legislative framework on data sharing that focuses on maximizing utility while also preserving privacy could be a major accelerating factor in fostering a data-driven culture. Establishing trust between government authorities and individuals would eventually create a conducive environment that promotes data sharing for the benefit of the public health system as a whole.

### Methodology

The scope of the review and the corresponding search strategy were intentionally broad to allow us to explore a relatively new concept that is not yet well defined. The term policy analytics, although present in the literature, was rarely used in the studies reviewed here. Instead, authors used separate terms to refer to (1) the analytics and tools designed (eg, data integration, aggregation, visualization, or analysis; BDA; platform; data-driven; evidence-based; and system) and (2) the use or purpose of the analytics (eg, policy [decision] making, [precision] public health, surveillance, monitoring, and population health). There are differences in the way the applications of tools are reported in the literature. For example, the term “use case” may refer to the actual use of the tool in a particular setting or scenario and examples of how the tool can be used. Various terms are also used to refer to conditions (eg, NCDs, public health, or the name of the individual condition) and end users (eg, policy makers, decision makers, and public health professionals). This highlights the difficulty to comprehensively review all such tools and their applications.

A quality assessment of the included studies was not performed in this review. Quality assessment is an optional step for scoping reviews. Even though it would provide a more robust evaluation of the evidence and help identify any potential biases, it was not considered crucial given the aims of the review (we were not interested in the actual data collected or the findings). However, the different types, aims, and scopes of the included studies have been clearly presented in the Study Characteristics subsection in the Results section.

Finally, we only searched 2 databases compared to other scoping reviews that included more sources of information. However, we selected 2 reliable databases that together cover both the medical and technological fields of literature and are therefore sufficient to explore the concept of data analytics tools and public health policies. The search strategy as a whole ensures the quality of the studies reviewed.

### Conclusions

There are many digital tools incorporating descriptive and predictive analytics that can support policy making in different ways for a range of NCDs. However, the majority of these tools are not widely used by policy makers in real-life. We have discussed factors affecting the adoption of digital health technologies as a whole and have also made recommendations on how stakeholders involved in the development and use of data analytics tools for public health policy making can help increase adoption and maximize the impact of such tools in supporting the policy-making process. The effectiveness and actual usefulness of the tools reviewed in this study should be assessed on the basis of the specific policy needs, settings, populations, conditions, and data with which they were tested.

## Appendix

Multimedia Appendix 1 PRISMA (Preferred Reporting Items for Systematic Reviews and Meta-Analyses Extension for Scoping Reviews) checklist.

## Abbreviations

AI: artificial intelligence
BDA: big data analytics
CRC: colorectal cancer
EHR: electronic health record
EU: European Union
MDPH: Massachusetts Department of Public Health
ML: machine learning
NCD: noncommunicable disease
